# Fetal Alcohol Spectrum Disorder: Can We Change the Future?

**DOI:** 10.1111/acer.14317

**Published:** 2020-03-30

**Authors:** Svetlana Popova, Danijela Dozet, Larry Burd

**Affiliations:** ^1^ Institute for Mental Health Policy Research Centre for Addiction and Mental Health Toronto ON Canada; ^2^ Dalla Lana School of Public Health University of Toronto Toronto ON Canada; ^3^ Factor‐Inwentash Faculty of Social Work University of Toronto Toronto ON Canada; ^4^ Faculty of Medicine Institute of Medical Science University of Toronto Toronto ON Canada; ^5^ Pediatric Therapy Services Altru Health System Department of Pediatrics North Dakota Fetal Alcohol Syndrome Center University of North Dakota School of Medicine and Health Sciences Grand Forks ND USA

In this issue of *Alcoholism: Clinical and Experimental Research,* 3 additional papers examining the prevalence of fetal alcohol spectrum disorders (FASDs; May et al., 2020a, 2020b, 2020c) follow the earlier paper by Chambers and colleagues (2019). These studies present state‐of‐the‐art FASD active case ascertainment (ACA) of prevalence among school‐age children conducted in the United States and demonstrate the importance of the behind‐the‐scenes intensive fieldwork necessary to complete studies of this size in community settings. The abundant clinical detail for each case is evident in the data on maternal and child characteristics presented in the papers. These studies confirm that FASD is a highly prevalent disorder in the U.S. general population.

In all 4 papers combined, 2,189 subjects were selected for assessment and 196 met criteria for FASD, including fetal alcohol syndrome (FAS; *n* = 25, 12.8%), partial FAS (*n* = 82, 41.8%), and alcohol‐related neurodevelopmental disorder (*n* = 89, 45.4%). These data show that approximately 1 in every 13 children with FASD had FAS. The data also improve our understanding of the lower threshold of teratogenic exposure, which increases the risk for FASD. Chambers and colleagues have found the threshold to be very low, showing 1 drink per drinking day during pregnancy to increase the risk of FASD by over 3‐fold (Chambers et al., 2019).

ACA studies are the gold standard for measuring prevalence of FASD in populations. Findings from ACA studies capture true population‐based prevalence, which illustrate the magnitude of the burden of FASD. Research shows that individuals with FASD have extensive impairments in development and high rates of mental health disorders and other comorbidities (Popova et al., 2016a, 2016b; Weyrauch et al., 2017) and thus experience many adverse health and social outcomes. As a result, FASD is expensive on the systemic level, posing an enormous burden to service systems as a result of healthcare utilization, special education, law enforcement, children in care, and productivity losses due to morbidity and premature mortality (Greenmyer et al., 2018; Popova et al., 2016). Fifty years after the term FAS was first introduced into the medical lexicon by Jones and Smith (1973), where are we at now?

## Screening and Diagnosis

Despite the availability of FASD diagnostic criteria and services in specialized clinics in such countries as Canada and the United States, a huge discrepancy exists between current prevalence estimates and community rates of diagnosis. FASD screening and diagnosis may not be accessible in some communities, and as such, it is largely misdiagnosed or undiagnosed (see, e.g., Chasnoff et al., 2015). Of the children born each year with FASD globally, we estimate that less than 1% will ever get a diagnosis (Burd and Popova, 2019). If early diagnosis and intervention are important to improve health and social outcomes, we are making painfully slow progress on the essential step of early identification. Why? The diagnostic criteria are too complex, resource‐ and service‐intensive, and they are age‐restrictive. Typically, a referral for an FASD assessment and diagnosis requires prior determination of prenatal alcohol exposure. The specific criterion for the assessment of exposure is determined by each clinic, but this information may be difficult to access in many cases.

In the United States, most diagnostic clinics are affiliated with universities and have astoundingly low diagnostic capacity. In the United States alone, we need to be able to evaluate 800 cases per day (live births multiplied by a conservative 5% prevalence) just to provide assessment of the estimated annual cohort of 200,000 cases of FASD per year.

In Canada, the capacity of FASD clinics is also low and the expertise needed to accommodate the demand in both countries is lacking. A 17‐fold increase in FASD diagnostic capacity across Canada is needed in order to diagnose existing FASD cases based on an outdated estimate of only 1% FASD prevalence (Clarren et al., 2011). In Canada and the United States, there are 4.3 million births per year. Using a conservative 5% FASD prevalence estimate, there are an estimated 9 to 10 million people with FASD born since the criteria for FAS were first reported 46 years ago. If we want to expand our diagnostic reach to this existing cohort from birth to 46 years of age, we need to add more capacity. If we plan to do it over the next 10 years, in the United States and Canada alone, we need to assess an additional 1 million people with suspected FASD per year. We must be clear: It is simply not feasible to see the millions of people with suspected FASD in the currently existing multidisciplinary neurodevelopmental clinics in the United States and Canada. Globally, the problem is far more difficult and the observation remains that only an estimated 1 in 600 people with FASD will ever be seen in one of these clinics. The current state of diagnostic capacity is such that the majority of FASD cases will never get a formal diagnosis; however, we still need to be able to provide informed and appropriate care for individuals with FASD in the community. Moreover, it is not clear that the current available criteria in Canada and the United States are fully representative of FASD in this larger population. We still utilize diagnostic criteria targeted toward young children, which are not readily applicable to infants, adults, or the elderly. To make diagnosis across the life span even more complex, the expression of FASD varies from person to person and also changes over the life span. Ideally, there would be one universal set of FASD diagnostic guidelines used in all countries that represent FASD across the life span. At the very least, we need changes in diagnostic criteria for FASD that rely less heavily on experienced dysmorphologists and medical geneticists. These clinicians, who have made the majority of all FASD diagnoses globally, are very small in numbers, and they have little additional diagnostic capacity.

How can we move to increase access to FASD diagnostic services, and improve early intervention and diagnosis‐informed care across the life span? We need brief, office‐based screening and diagnosis for FASD. Current, ongoing developments may be promising; many hope that advances in Web‐based technology can make screening and diagnosis more accessible. For example, telehealth has successfully been used for FASD assessment in rural and remote communities in Manitoba since 2000 (Hanlon‐Dearman et al., 2014). Computer‐based facial recognition software has been shown to have high diagnostic accuracy for detecting ARND, perhaps even higher than standard, manual assessment methods (Valentine et al., 2017). Additionally, a new cost‐effective screening tool using a camera and computer vision to track eye movement has been shown to effectively screen for FASD in young children, in only 10 to 20 minutes (Zhang et al., 2019). Progress is being made in early diagnosis with some data suggesting that a diagnosis can be made as early as at 9 months (Kalberg et al., 2019). Some form of clinical appraisal to allow the implementation of diagnosis‐informed care is essential for the majority of people with FASD. Diagnosis is essential, and it is the first step in reducing exposure to future adverse experiences and providing appropriate support services. If an individual needs a more extensive assessment, community clinicians can then refer perhaps as many as 2 to 3% of complex cases needing specialized services to multidisciplinary clinics for diagnosis.

Currently, FASD is not widely recognized by healthcare practitioners. In many countries, almost no cases are diagnosed due to limited FASD awareness and a lack of diagnostic training provided to clinicians. We must introduce mandatory training on FASD screening, diagnosis, and treatment for healthcare providers, psychiatrists, psychologists, social workers, individuals working within the justice system, and other professionals. As they typically encounter individuals with FASD, this training would result in an improved referral rate for suspected cases and increased access to appropriate services.

Prevalence rates of FASD vary between and also within countries. FASD is known to be substantially more prevalent in certain sub‐populations globally, such as correctional populations, children in care, and specialized clinical and special education populations (Popova et al., 2019). It is imperative to actively screen these sub‐population high‐risk groups for prenatal alcohol exposure and FASD to address population‐specific needs and improve overall outcomes.

Furthermore, nothing limits our understanding of FASD prevalence and symptomatology across the life span more than the lack of a distinctive diagnostic code in the International Classification of Diseases (ICD) and in the American Psychiatric Association Diagnostic and Statistical Manual (DSM). It is crucial to continue to advocate for the harmonization of the ICD and DSM diagnostic criteria for FASD.

Reducing stigma associated with prenatal alcohol exposure and FASD is another essential priority in order to improve data collection and to provide adequate diagnostic and treatment services. Birth mothers of children with FASD are more likely to experience stigma and discrimination compared to women with mental illness, previous involvement with the legal system, or women with substance use disorders (Corrigan et al., 2017). In order to reduce this stigma that can negatively impact the patient, birth mother, and family members, we should explore the use of an alternative diagnostic term for FASD that does not specify fetal alcohol exposure. An alternative term could minimize barriers to women’s self‐report of alcohol consumption during pregnancy and, therefore, lead to more determination of prenatal alcohol exposure, which could then result in earlier diagnosis and intervention.

We must improve our capacity for early diagnosis of FASD in order to facilitate early intervention and the application of diagnosis‐informed risk reduction programs to improve health and social outcomes across the life span in this population. Ongoing research in developing specific biomarkers of prenatal exposure and/or effects may yield novel means in screening and diagnosis.

## Prevention

Globally, the prevalence of alcohol consumption during pregnancy is 9.8% (Popova et al., 2017). Various published national guidelines emphasize that the safest option is to abstain from alcohol during pregnancy. However, some countries suggest that low levels of alcohol consumption during pregnancy may also be safe (Furtwaengler and de Visser, 2013). We need to disseminate a universal message of “zero alcohol use during pregnancy or when trying to become pregnant” and to provide education about the detrimental consequences of alcohol use during pregnancy, including FASD.

Several studies have found brief interventions for alcohol use in pregnant women to be effective in increasing alcohol abstention rates and minimizing alcohol consumption levels (O'Connor et al., 2018). It is critical to establish universal screening for alcohol use and to provide brief interventions to all childbearing age and pregnant women in general and, especially high‐risk populations, as appropriate. Upon pregnancy recognition or confirmation, the majority of pregnant women report quitting drinking or at least reducing alcohol intake (McCormack et al., 2017); however, an estimated 44% of pregnancies globally were unintended from 2010 to 2014 (Bearak et al., 2018), which may delay pregnancy recognition and increase the risk of fetal alcohol exposure.

Often, prenatal alcohol exposure and FASD recur within the same family (Burd and Popova, 2019); this is escalated by the fact that birth mothers of children with FASD have higher levels of prepregnancy and postnatal alcohol consumption and are more likely to have FASD themselves (McQuire et al., 2019). Therefore, it is important to provide postpartum support to new mothers, especially mothers of children with FASD, in order to prevent the recurrence of FASD in these families.

Furthermore, in prevention efforts, we must attend to the roles of many risk factors such as prenatal polysubstance exposures (e.g., tobacco, marijuana, opiates, cocaine, and prescription medications), sociodemographic, gestational, nutritional, and genetic factors in the FASD causal chain (Behnke and Smith, 2013; McQuire et al., 2019).

Adverse childhood experiences (ACEs), such as trauma, need to be understood in the context of preventing alcohol consumption during pregnancy and FASD. Women with ACEs are more likely to consume alcohol during pregnancy, and individuals with FASD are more likely to have ACEs, which contributes to the development of comorbid disorders in FASD (Kambeitz et al., 2019; Weyrauch et al., 2017). Understanding the important role of FASD as a predisposing risk marker for ACEs could reduce the large economic burden placed on service systems (Kambeitz et al., 2019), could improve outcomes for diagnosed individuals, and could possibly prevent FASD familial recurrence.

There is an urgent need to address prepregnancy alcohol use patterns in women of childbearing age. Alarmingly, prevalence rates of alcohol consumption and binge drinking among women of childbearing age have been increasing globally (World Health Organization, 2018) due to a number of factors, including economic development, increased availability and accessibility of alcohol, social acceptability of drinking among women, shifting gender roles, and alcohol marketing targeting women of childbearing age.

This demonstrated trend of increased alcohol consumption not only poses a risk to women’s health, but also increases the chances of pregnancies to become alcohol‐exposed. Several population‐based policy options exist that aim to reduce alcohol use among populations, including women of childbearing age and pregnant women. These measures are effective and cost‐effective in reducing the risk of alcohol‐attributable disease burden, including FASD, and are listed in the *WHO Global strategy to reduce the harmful use of alcohol* (World Health Organization, 2010) and in *the WHO Global action plan for the prevention and control of non‐communicable diseases 2013 to 2020* (World Health Organization, 2013), and should be adopted by all countries.

## Treatment and Management

Early diagnosis is crucial in providing timely interventions and support services to people with FASD and their families. Although several types of interventions, including behavioral, pharmacological, nutritional, and environmental exist, we need increased evidence on new and more effective treatment methods that take into account the range of adverse experiences and comorbid conditions experienced by individuals with FASD.

Current research and clinical findings on the experiences of individuals with FASD are based solely on diagnosed cases, which are a small minority (less than 1% of all cases; Burd and Popova, 2019) and it is not clear that these cases are representative of the entire population with FASD. As more individuals are diagnosed and research is conducted on additional sub‐populations, we will obtain a greater understanding of the complexity of FASD across the life span, including symptomatology, comorbidities, and the range of support services needed.

Additionally, substantial changes to current practice may be needed for the successful diagnosis and management of FASD in infant, adult, and elderly populations. The domains of impairment in FASD change across the life span. This suggests that FASD may not be adequately assessed and diagnosed at one specific point in time and that a single diagnosis may not be optimal for the management of the disorder across the life span. People with FASD will likely need follow‐up in their community to address their individualized needs. Additionally, sentinel facial features in individuals with FASD may fade over time, rendering this disorder truly invisible in the adult and elderly populations and heightening the need for access to diagnostic and case management services.

Adequate collection of data and information about FASD and prenatal alcohol exposure in high‐risk groups is needed to advocate for the appropriate treatment and management of FASD in these sub‐populations. One approach might be the development of centralized surveillance systems or registries for FASD and prenatal alcohol exposure, in order to monitor trends over time and to evaluate the effectiveness of prevention initiatives and interventions.

How could we afford this? We now have a potential model of how to target our funding for FASD prevention (Greenmyer et al., 2019). If we emphasize the implementation of prevention activities in high‐risk subpopulations, we are likely to enhance the impact of our FASD prevention funding. Strategically implementing national funding for FASD prevention can have tremendous benefits, as it is known to be over 1,000 times more cost‐effective to identify a woman who has already has a biological child with FASD, than to implement interventions with women of childbearing age who consume alcohol (Greenmyer et al., 2019).

There are over 10 million affected people just in the United States and Canada alone. It is clear that we are currently spending billions on FASD annually; most of this money is spent on treating people with FASD for something else (i.e., incorrect or missing diagnosis), and only a small portion of it is directed toward research and prevention. We are missing out on the benefits of early diagnosis and diagnosis‐informed care, but how could we afford this? Currently, 10 million people with FASD multiplied by the annual cost of care ($22,810 for each child and $24,308 for each adult annually) suggests billions are spent each year, yet only a small fraction of this money would be required to provide accurate community‐based assessment, diagnosis‐informed interventions, and most importantly, prevention initiatives.

To conclude, after 50 years of research and clinical practice in FASD, we have created an overwhelming evidence base to suggest implications for FASD prevention and support services in light of trends in alcohol use during pregnancy and known outcomes for individuals with FASD. While many diagnostic, prevention, and intervention services do exist, there is far more that is yet to be done on the national and global scales.

## Conflict of interest

The authors declare they have no financial or nonfinancial competing interests relevant to this article to disclose.
